# Implementation preferences for the management of sexually transmitted infections in the South African health system: a discrete choice experiment

**DOI:** 10.1136/sextrans-2023-055816

**Published:** 2023-11-02

**Authors:** Collins Iwuji, Catherine E Martin, Diantha Pillay, Patience Shamu, Susan Nzenze, Mercy Murire, Laura Ashleigh Cox, Alec Miners, Carrie Llewellyn, Saiqa Mullick

**Affiliations:** 1 Department of Global Health and Infection, Brighton and Sussex Medical School, University of Sussex, Brighton, UK; 2 Africa Health Research Institute, Durban, South Africa; 3 Department of Implementation Science, Wits Reproductive Health and HIV Institute, Johannesburg, South Africa; 4 Faculty of Public Health and Policy, London School of Hygiene and Tropical Medicine, London, UK; 5 Primary Care and Public Health, Brighton and Sussex Medical School, University of Sussex, Brighton, UK

**Keywords:** HIV pre-exposure prophylaxis, SYNDROMIC MANAGEMENT, PARTNER NOTIFICATION, Bacterial Infections, SEXUAL HEALTH

## Abstract

**Objectives:**

Despite strengthening HIV prevention with the introduction of pre-exposure prophylaxis (PrEP), STI services have remained relatively unchanged and the standard of care remains syndromic management. We used a discrete choice experiment to investigate service users’ preferences for the diagnosis and treatment of STIs in South Africa.

**Methods:**

Between 1 March 2021 and 20 April 2021, a cross-sectional online questionnaire hosted on REDCap was administered through access links sent to WhatsApp support groups for HIV PrEP users and attendees of two primary healthcare clinics and two mobile facilities in the Eastern Cape and Gauteng provinces aged between 18 and 49 years. Participants either self-completed the questionnaire or received support from a research assistant. We used a conditional logit model for the initial analysis and latent class model (LCM) to establish class memberships, with results displayed as ORs and probabilities.

**Results:**

We enrolled 496 individuals; the majority were female (69%) and <30 years (74%). The LCM showed two distinct groups. The first group, comprising 68% of the participants, showed a strong preference for self-sampling compared with no sampling (OR 2.16, 95% CI 1.62 to 2.88). A clinic follow-up appointment for treatment was less preferable to same-day treatment (OR 0.78, 95% CI 0.63 to 0.95). Contact slip from index patient (OR 0.86, 95% CI 0.76 to 0.96) and healthcare professional (HCP)-initiated partner notification (OR 0.63, 95% CI 0.55 to 0.73) were both less preferable than expedited partner treatment (EPT). The second group included 32% of participants with a lower preference for self-sampling compared with no sampling (OR 0.65, 95% CI 0.41 to 1.04). There was no treatment option that was significantly different from the others; however, there was a strong preference for HCP-initiated partner notification to EPT (OR 1.53, 95% CI 1.10 to 2.12).

**Conclusions:**

Our results suggest that service users preferred STI testing prior to treatment, with the majority preferring self-taken samples and receiving aetiology-based treatment on the same day.

**WHAT IS ALREADY KNOWN ON THIS TOPIC**

- STI care in sub-Saharan Africa is based on the presence of symptoms, which is known as syndromic management.
- Evidence is needed to inform transition from syndromic management to treating the actual cause of STI and integrating this approach within existing health services.

**WHAT THIS STUDY ADDS**

- Our study showed that service users prefer to undergo STI testing prior to treatment, with the majority preferring self-taken samples and same-day treatment.
- Service users also prefer to be given a choice for partner notification, with the majority preferring to take the medications to their partners.

**HOW THIS STUDY MIGHT AFFECT RESEARCH, PRACTICE OR POLICY**

- This study provides evidence of services users’ preference for the diagnosis and treatment of STI within primary care clinics in South Africa.
- The identified STI care strategies will need to be evaluated in implementation studies.

## Introduction

STIs present a major global public health problem, especially in women. While STIs affect individuals of all ages, adolescents and young people are disproportionately affected.^1^ High rates of STIs have been identified among young people in sub-Saharan Africa, with South African household studies reporting prevalence rates of any curable STI of 14% among those aged 15–24 years old,^2^ and prevalence rates of *Neisseria gonorrhoeae* (NG) of 3.2% and 6.5% and of *Chlamydia trachomatis* (CT) of 11.7% and 15.6%.^2 3^ The prevalence and incidence of STI are also particularly high among young women receiving HIV pre-exposure prophylaxis (PrEP),^1 4^ with a meta-analysis reporting chlamydia and gonorrhoea prevalence of 15.1% and 4.6% among women aged 15–24 years and 7% and 2.5% in those aged 25–49 years, respectively, in South Africa.^1^

Although the scale-up of PrEP and the provision of an integrated approach to HIV prevention and sexual and reproductive health (SRH) provide an opportunity to improve STI control,^5^ STI diagnosis and management services in sub-Saharan Africa have remained relatively unchanged.

The current standard of care in South Africa remains syndromic management in which individuals presenting with STI symptoms receive treatment based on national algorithms.^6^ The drawback of this approach is that not all symptoms suggestive of STIs are actually due to STIs. For example, in a South African STI surveillance study, the most common causes of vaginal discharge syndrome were bacterial vaginosis and candidiasis,^7^ while 19% of clinic presentations with vaginal discharge did not have any detectable STI or non-STI cause in another study.^8^ Furthermore, the majority of people with STIs are asymptomatic, hence will remain undiagnosed and untreated using the syndromic approach.^2 9 10^

The WHO has identified better diagnosis, treatment and partner services for populations at high and ongoing risk of acquiring STIs as key strategies to achieving global targets to ending STI epidemics as public health concerns by 2030.^5^ The South African National Strategic Plan for HIV, tuberculosis and STIs (2017–2022) highlights the need to improve the detection and management of asymptomatic STIs with increased laboratory support and use of point-of-care (POC) testing,^11^ and national STI management guidelines make provision for the implementation of asymptomatic STI screening strategies for adolescent girls and young women accessing SRH services.^6^ However, the implementation of these approaches is limited.

Evidence is needed to inform the revitalisation of STI services in sub-Saharan Africa and the transition from syndromic STI care to an aetiology-based approach that takes advantage of the recent advances in STI diagnostics and effectively integrates these within existing health services.^12 13^

We aimed to ascertain health service users’ preferences for diagnosis and treatment of STIs, receipt of results, treatment and partner notification (PN) using discrete choice experiments (DCEs). We hypothesised that service users would prefer an STI care model that is designed to identify the aetiology of their symptoms and provide same-day treatment at the shortest waiting time.

## Methods

### Study setting

The Wits Reproductive Health and HIV Institute implements an HIV PrEP implementation science project in four clusters in South Africa. Two of the clusters were identified as sites for this study. The study included individuals receiving PrEP or other SRH services at the fixed and mobile units of urban and periurban primary healthcare clinics in Tshwane subdistrict in Gauteng Province and Nelson Mandela Bay in Eastern Cape Province of South Africa, respectively, using a combination of unassisted and research assistant-supported online surveys. These two districts have high prevalence of HIV and STIs.^14–16^

### Study design

The study employed an analytic cross-sectional design with a DCE. We used a DCE to enable us to quantify the strength of the STI service preferences and trade-offs. The DCE is grounded in random utility theory (RUT), an economics-based theory which postulates that individuals make rational decisions and that goods are described by their characteristics.^17^ Further RUT postulates that individuals are likely to select a service based on maximum utility. Using DCE allows for potential service users to state their preferences given hypothetical scenarios, goods or services that are presented to them even when the options are not yet available.^18^ The responses given are then used to infer the value placed on each attribute.

Participants were required to complete a questionnaire in which they were asked to imagine that they were being offered different options for STI care during a clinic attendance prior to answering a series of questions (online supplemental table S1) in which they were required to choose service preferences offered by either clinic A or clinic B. The options were described according to a number of characteristics (known as ‘attributes’), such as how and when treatment for the STI is given, and within each attribute are levels.

10.1136/sextrans-2023-055816.supp1 Supplementary data

The study is reported according to the International Society for Pharmacoeconomics and Outcomes Research Good Research Practices for Conjoint Analysis.^19^

### DCE instrument design

#### Choice of attributes and levels

The DCE attributes relating to STI sample collection, diagnosis, receipt of results, receiving treatment and notification of partners and the levels within each attribute were informed by a review of relevant literature^10 12 13 20 21^ and discussion with experts. This informed the study matrix of attributes and levels as described in table 1 for each service delivery consideration (attributes). Attributes were considered as the characteristics of a service delivery model for STI care and treatment. There were four attributes, and each had three to four ‘levels’ (table 1). The levels are the value of scenarios that each attribute can take on. The levels assigned represent both standard of care and alternatives listed in the literature and these were refined through multiple discussions with individuals who may represent the demographic of the participants, study team/collaborators, methodologists, experts in the field, and relevance to the research question and policy context.

**Table 1:** Discrete choice experiments attributes and levels

Attributes	Levels	Description
How the STI testing sample is taken	No sample is taken; care is based only on presenting symptoms.	No samples are taken; you are given treatment just based on whether you have symptoms or not.
	You self-sample at the clinic.	There are different types of samples: urine for men and vaginal swab for women. After being provided with instructions, you could take the sample yourself at a private place at the clinic.
	By a healthcare professional at the clinic.	The provider takes a sample when examining you.
How and when you are told if you have an STI	The same day after a 2-hour wait at the clinic by a healthcare professional.	This may be the shortest time you will spend in the clinic in total because diagnosis is based on symptoms only; no samples are taken, so the provider will not know whether you really have an infection or not and will also not know which infection/s you have.
	The same day after a 4-hour wait at the clinic by a healthcare professional.	You will spend a longer time in the clinic because samples will be taken and tested at the clinic, so you will definitely know if you have an infection and what it is.
	In 1–7 days’ time via a secure online website.	You may spend about 2 hours in the clinic because samples will be taken but it will not be tested at the clinic; rather, they will be sent away for testing, so your results will not be available on the same day. You will be given a secure code at the clinic and a website address to check your results privately. If you have an infection, you may still have to go back to the clinic for treatment.
	In 1–7 days’ time via text message (SMS).	You may spend about 2 hours in the clinic because samples will be taken but it will not be tested at the clinic; rather, they will be sent away for testing, so your results will not be available on the same day. You will receive a text message when your results are ready, which will either say ‘Your Results Are Ready – No Follow-up Visit Required’ or ‘Your Results Are Ready – Please present at your preferred clinic as soon as possible’. If you have an infection you will have to go back to the clinic for treatment.
How and when treatment for your STI is given if required	From a place near you, such as a local pharmacy.	Medication can be picked up from a local pharmacy.
	At the clinic during a follow-up appointment.	If you receive an SMS requiring you to revisit the clinic or if the online system indicates you have an infection, you will be required to come back to the clinic to collect your treatment.
	At the clinic the same day.	If you are being treated according to your symptoms or not or if you are being tested and the test is being done at the clinic on the same day.
How your partner(s) is/are notified if you have an STI	You notify your partner using a notification slip.	We will give you a slip to take to your partner/s. The slip will inform your partner to attend the clinic for diagnosis and/or treatment.
	Your partner(s) is/are notified directly by a healthcare professional.	If you prefer, the provider can contact your partner(s) and invite them to the clinic for testing and treatment, without mentioning you.
	Expedited partner treatment.	If you are being treated for an infection, you will be given treatment to give to your partner(s) for the same infection.

SMS, short messaging service.

There were a total of 108 possible combinations of the STI attributes and levels that were selected for the study (3×3×3×4), resulting in 5778 total combinations (108×107/2) across two scenarios (A or B). This number of combinations is too many to present to the participants; hence, the DCE instrument was designed using a D-efficient approach with 12 choice tasks (questions) using Ngene software,^22^ ensuring that preferences for each of the attribute levels could be independently assessed. A questionnaire was also administered to collect STI risk factors and sociodemographic information (online supplemental table S1).

### Procedures and data collection

The DCE questionnaire was administered between 1 March 2021 and 20 April 2021. Piloting of the study recruitment and data collection processes was undertaken prior to study implementation and informed the final recruitment and data collection strategies. Participants aged 18–49 years old were recruited from study sites using the following approaches:

#### Direct link to the questionnaire received by the participants

Potential participants accessed or received a link to the online questionnaire through the clinic’s WhatsApp groups (support groups for PrEP users moderated by peer navigators). The online questionnaire was created on a Research Electronic Data Capture (REDCap) database hosted on the University of Witwatersrand website.^23^ A recorded voice message guided the participants through the study rationale and informed consent process, DCE attributes and levels with explanation and pictorial illustrations, and the questionnaire (online supplemental figure S1).

#### Recruitment at the healthcare facility

Individuals attending either the mobile or fixed clinic facilities at the study sites were recruited to the study by a research assistant, who described the study rationale, obtained informed consent, explained the DCE attributes and levels, and then supported the participant to complete the study questionnaire.

Regardless of the approach used for participant recruitment, there were two methods of data collection: (1) online questionnaire completion without research assistant support, henceforth referred to as data collection (DC)1; and (2) online questionnaire completion with research assistant support, henceforth referred to as DC2.

All participants received the equivalent of US$14 in the local currency, provided as airtime vouchers.

Determining sample sizes in advance of conducting DCEs is difficult because the questionnaire design is usually not known at the study’s outset. However, as it has been suggested that a reasonable sample size is 300, we aimed to recruit at least 500 participants with complete answers to allow us to examine the predictors of group membership in a planned latent class modelling.^24^

### Statistical analysis

Descriptive characteristics of the study participants were tabulated according to the data collection method used. The results are presented as ORs relative to the relevant base category and show the probabilities of uptake of any of the other categories. Reported SEs are adjusted in all instances to account for the potential clustering in participant responses.

We used the conditional logit (CLOGIT) model to analyse the DCE.^25^ Unlike in standard logistic regression, the results from CLOGIT models are ‘conditional’ on the information relating to all the choice options as this information is grouped before the analysis. In this sense, CLOGIT models are akin to matched case–control designs as they investigate the relationship between a choice (case), options that were not chosen (controls) and a set of predictive factors (attribute levels).

Although the CLOGIT model is recommended to be used for the initial analysis,^26^ it produces results for the ‘average’ individual, meaning that no allowance is made for the possibility that different groups of people within the sample (eg, different age groups) might have varying preferences—this is known as ‘preference heterogeneity’. Hence, we also analysed the results using a latent class model (LCM) as it simultaneously relaxes the independence of irrelevant alternatives assumption and allows potential preference heterogeneity to be examined.^27^ LCMs are recommended if groups of respondents with similar preferences are anticipated.

LCMs assume there are subgroups of individuals (classes) with similar preferences and that the likelihood of class membership can be related to observed variables. The potential predictors of class membership in this analysis were gender, age (as a categorical variable), current STI symptoms (yes, no), previous STI treatment (yes, no), facility location (urban, periurban, other), employment (not employed, part-time or full-time employment, student) and method of data collection (no research assistant support, research assistant support provided). To determine the optimal number of classes to include in the LCM, we estimated models with two to five classes and the number of classes in the final LCM was based on minimisation of Akaike’s information criterion and the production of stable/meaningful SEs. The CLOGIT and LCM analyses were undertaken using Stata V.16 and NLOGIT V.5 (Econometric Software, Plainview, New York), respectively.

## Results

### Characteristics of participants

The online questionnaire link for DC1 (no assistance provided for questionnaire completion) was completed by 291 participants. Of the 213 individuals who received support to complete the online questionnaire (DC2), 205 provided consent and completed the full survey. In total, 496 individuals completed the questionnaire across the two data collection methods. About 367 (74%) participants were <30 years old, with a median of 25 years (IQR 22–29), and there was no age difference between the two groups by method of data collection. The majority were female (69%), with no gender difference by method of data collection. There was evidence of high-risk sexual behaviour among the recruited participants, with 144 (29%) having previously been treated for STIs and 101 (20%) reporting current STI symptoms. Those in DC2 were more likely to have reported their employment status as students (p<0.001), less likely to have reported condomless sex in the last year (p<0.001) and more likely to be users of the clinic facilities selected for recruitment of participants (p<0.001) than those in DC1 (table 2).

**Table 2:** Sociodemographics and sexual risk behaviour by data collection methods

Variables	Data collection 1 (self-completion) n=291 (%)	Data collection 2 (completion with support) n=205 (%)	P value
Age (years), median (IQR)	26 (22–29)	24 (21–28)	0.134
18–24	117 (40.2)	113 (55.1)	
25–29	90 (30.9)	47 (22.9)	
≥30	68 (23.4)	45 (22.0)	
Missing	16 (5.4)	–	
Gender			0.145
Male	92 (31.6)	57 (27.8)	
Female	195 (67.0)	148 (72.2)	
Missing	4 (1.4)	–	
Employment status			<0.001
Not employed	185 (63.6)	104 (50.7)	
Employed	86 (29.6)	46 (22.4)	
Student	17 (5.8)	55 (26.8)	
Missing	3 (1.0)	–	
Ever had sex under the influence of alcohol or drugs			0.993
Yes	95 (32.6)	67 (32.7)	
No	196 (67.4)	138 (67.3)	
Condomless sex in the last year			<0.001
Yes	190 (65.3)	166 (81.0)	
No	101 (34.7)	39 (19.0)	
Current STI symptoms			0.534
Yes	62 (21.3)	39 (19.0)	
No	229 (78.7)	166 (81.0)	
Previous STI treatment			0.923
Yes	84 (28.9)	60 (29.3)	
No	207 (71.1)	145 (70.7)	
Current sexual partners			0.174
0	56 (19.2)	25 (8.6)	
1	209 (71.8)	156 (76.1)	
2–4	22 (7.6)	21 (10.2)	
>4	4 (1.4)	3 (1.5)	
Clinic used for care			<0.001
Tshwane: urban	63 (21.6)	131 (63.9)	
Nelson Mandela Bay: periurban	67 (23.0)	73 (35.6)	
Outside study clinics	161 (55.3)	–	
Missing	0	1 (0.4)	

### STI care preferences

#### Preference of the ‘average’ individual

The CLOGIT model produced results showing the STI preferences of the ‘average’ individual (table 3). This showed a strong preference for self-sampling (OR 1.47, 95% CI 1.27 to 1.71), with no significant difference between healthcare professional (HCP) sampling and no sampling. There was a much lower preference for having to wait 4 hours in the clinic to get the results on the same day compared with a 2-hour wait (OR 0.82, 95% CI 0.73 to 0.92), with the latter being no different to getting the results in 1–7 days by either SMS (short messaging service) or secure online portal. There was a slightly lower preference for a clinic follow-up for treatment compared with same-day treatment (OR 0.91, 95% CI 0.81 to 1.01, p=0.077), with preference for the latter being no significantly different to treatment at a local pharmacy. There was a lower preference for PN by the index patient (OR 0.93, 95% CI 0.87 to 0.99) or provider-initiated (OR 0.83, 95% CI 0.77 to 0.89) compared with expedited partner treatment (EPT).

**Table 3:** STI testing preferences of the ‘average’ individual

Variables	OR (95% CI)	P value
How STI samples should be taken		
No sampling	1	
HCP	0.99 (0.90 to 1.09)	0.845
Self-sampling	1.47 (1.27 to 1.71)	<0.001
How and when results are given		
Same day after 2 hours	1	
Same day after 4 hours	0.82 (0.73 to 0.92)	0.000
1–7 days’ time via SMS/online	1.05 (0.95 to 1.16)	0.301
How and when treatment is given		
Same day in clinic	1	
Clinic follow-up on appointment	0.91 (0.81 to 1.01)	0.077
Pharmacy near patient	1.06 (0.99 to 1.13)	0.110
How partners are notified of an STI		
Expedited partner treatment	1	
Contact slip from index patient	0.93 (0.87 to 0.99)	0.028
HCP-initiated notification	0.83 (0.77 to 0.89)	0.000

HCP, healthcare professional; SMS, short messaging service.

### Latent class model

The LCM identified two groups, with 68% and 32% of the participants likely to be in groups 1 and 2, respectively (table 4).

**Table 4:** STI testing preferences: results from the latent class model

Variables	Group 1 (68%) (self-led)		Group 2 (32%) (HCP-led)	P value
	OR (95% CI)	P value	OR (95% CI)	
How STI samples should be taken				
No sampling	1		1	
HCP	0.93 (0.79 to 1.09)	0.368	1.18 (0.88 to 1.57)	0.265
Self-sampling	2.16 (1.62 to 2.88)	<0.001	0.65 (0.41 to 1.04)	0.07
How and when results are given				
Same day after 2 hours	1		1	
Same day after 4 hours	0.63 (0.51 to 0.77)	<0.001	1.45 (1.05 to 2.00)	0.023
1–7 days’ time via SMS/online	1.03 (0.88 to 1.21)	0.689	1.07 (0.81 to 1.41)	0.640
How and when treatment is given				
Same day in clinic	1		1	
Clinic follow-up on appointment	0.78 (0.63 to 0.95)	0.012	1.27 (0.92 to 1.76)	0.149
Pharmacy near patient	1.16 (1.04 to 1.29)	0.006	0.86 (0.72 to 1.04)	0.121
How partners are notified of an STI				
Expedited partner treatment	1		1	
Contact slip from index patient	0.86 (0.76 to 0.96)	0.010	1.10 (0.92 to 1.32)	0.285
HCP-initiated notification	0.63 (0.55 to 0.73)	<0.001	1.53 (1.10 to 2.12)	0.011

HCP, healthcare professional; SMS, short messaging service.

Group 1 had a strong preference for self-sampling compared with no sampling (OR 2.16, 95% CI 1.62 to 2.88, p<0.001), with no significant difference between HCP sampling and no sampling (syndromic management). There was a much lower preference for a 4-hour wait for results compared with a 2-hour wait (OR 0.63, 95% CI 0.51 to 0.77, p<0.001), with the latter not being significantly different to getting the results in 1–7 days by either SMS or secure online portal. There was a lower preference for a clinic follow-up appointment for treatment than same-day treatment (OR 0.78, 95% CI 0.63 to 0.95, p=0.012). There was a strong preference for receiving treatment from a local pharmacy compared with waiting 2 hours for same-day treatment (OR 1.16, 95% CI 1.04 to 1.29, p=0.006). There was a much lower preference for PN by the index patient (OR 0.86, 95% CI 0.76 to 0.96, p=0.010) or HCP-initiated (OR 0.63, 95% CI 0.55 to 0.73, p<0.001) compared with EPT. We referred to group 1 members as those preferring a self-led service.

Group 2 had a lower preference for self-sampling compared with no sampling (OR 0.65, 95% CI 0.41 to 1.04, p=0.07), although the evidence for this was weak. There was no statistically significant difference between HCP sampling and no sampling. There was a strong preference for waiting 4 hours for same-day results compared with 2 hours (OR 1.45, 95% CI 1.05 to 2.00, p=0.023), with the latter not being significantly different to receiving results in 1–7 days by SMS/online. There was no statistical difference in preference for the three treatment options. There was a strong preference for PN by an HCP compared with EPT (OR 1.53, 95% CI 1.10 to 2.12, p=0.011) and no significant difference between PN by the index patient and EPT. We refer to group 2 participants as those preferring an HCP-led service.

Participants were more likely to prefer a self-led to an HCP-led service if they were aged 25–49 years compared with 18–24 years (p=0.001) and to receive care from a periurban rather than an urban facility (p=0.011). Employed individuals were more likely to prefer an HCP-led service to a self-led service (p=0.038). The other variables were not predictive of class memberships (online supplemental table S2).

## Conclusions

We conducted a DCE to investigate service users’ preferences for the management of STIs in primary healthcare facilities providing HIV PrEP. Our results showed that the ‘average’ individual prefers to self-sample, receive their results within 2 hours, may not want to come back to the clinic on a different day for treatment and would prefer EPT to other forms of PN.

However, the LCM revealed two groups of individuals with different STI service delivery preferences. About two-thirds of individuals, classified as preferring a self-led service, would prefer to self-sample, be informed of their results within 2 hours on the same day or 1–7 days later by SMS/online, receive treatment from a pharmacy that is local to them and use EPT. These individuals were more likely to be older than 25 years and receive care from periurban healthcare facilities.

The remaining one-third of individuals, classified as preferring HCP-led service, showed a lower preference for self-sampling, with no difference between HCP sampling and no sampling. They would be willing to wait up to 4 hours in a clinic to get their results, may not mind returning to the clinic for treatment and would prefer the HCP-led PN if required. These individuals were more likely to be younger than 25 years, employed and receive their care from urban healthcare facilities.

The South African PrEP guidelines recommend either syndromic or aetiology-based STI care in individuals at high risk of HIV acquisition who require PrEP. However, aetiology-based STI care is not routinely available in the public health programme except as part of implementation research in certain facilities. The service currently available for STI care in health facilities differs from the preferences of service users based on our study results.

Our results demonstrate that service users want to be tested for the presence of an actual STI prior to being treated. Other studies in South Africa have demonstrated that the self-taken swab is acceptable and feasible.^2 28^ In one of the studies, nearly two-thirds of individuals preferred self-taken swabs and the remaining one-third either preferred HCP-taken swabs or expressed no preference.^28^ We observed that those older than 25 years who receive their care from periurban clinics showed a preference for getting their results within 2 hours on the same day or by SMS in 1–7 days, with the latter choice being available to those who wanted an aetiology-based approach. There was a preference for same-day treatment or treatment from a local pharmacy, with a clinic follow-up being less preferred. This would suggest that an aetiology-based approach would be ideal for this group if results can be provided within 2 hours on the same day or substituted with the convenience of receiving treatment from a local pharmacy.

About one-third of those who participated in the DCE indicated they would prefer to wait for up to 4 hours if it meant that they received diagnostic testing for their STIs, with weak evidence of a preference to return to the clinic for treatment during a follow-up appointment. The likelihood of receiving same-day treatment would be high in this group as they were willing to wait 4 hours for aetiology-based diagnosis, which might be the reason for the lack of a statistical difference between the three treatment options presented. However, in a recent study in Zimbabwe, individuals 16–24 years old were unwilling to wait 90 min for their results when the GeneXpert CT/NG was used as an STI POC diagnostic tool.^29^

The two groups of individuals in this study differed in how they would want their sexual partners to be notified if they were diagnosed with an STI. Two-thirds of individuals had a strong preference for EPT, while a third preferred an HCP-initiated notification. EPT has been shown to be acceptable to South African women of different age groups and their partners^10 30^ and resulted in a decrease in STIs in a follow-up test among those who accepted EPT.^10^

This DCE suggests STI service users will prefer an aetiology-based STI management approach and models of STI care that allow them to obtain their results and treatment in a prompt and convenient manner. This would mean making available treatment options for aetiology-based STI care that include same-day clinic treatment as well as community treatment. The availability of POC STI technologies that provide results for CT, NG and *Trichomonas vaginalis* in 30 min^12 13^ puts this model of aetiology-based STI care within reach, potentially allowing an episode of care to be completed within 2 hours of the patient arriving in the clinic. Aetiology-based STI care has the advantage of detecting STIs in asymptomatic individuals who would otherwise remain undiagnosed and untreated. It could also prevent overtreatment of people with genital symptoms with antibiotics for STIs which they do not have, hence contribute to reducing the burden of antimicrobial resistance through both good antibiotics stewardship and reduction in STI burden.^31^

Our study has a few limitations. First, participants were presented with a hypothetical scenario in which they were asked to imagine they were having an STI test. It is possible that some participants could struggle to determine their preference for STI care (eg, self-testing, EPT) which they have never experienced; however, there is growing evidence that DCEs predict actual behaviour.^32 33^ It will be critical to evaluate these preferences in real-world implementation studies. Second, our study recruited individuals with access to mobile phones; hence, our results may not be generalisable to those individuals who may not easily access a mobile phone.

The strengths of the study include the recruitment through WhatsApp, social media, and periurban and urban settings through fixed and mobile clinics in the community, which meant we were able to reach individuals with varied sexual risk behaviours. Our sample size is fairly large for a DCE, of which the recommended sample size threshold is around 300.^24^

The DCE suggests that service users prefer aetiology-based STI care but differ in how this should be made available. This highlights the need for a range of STI care options as one size does not fit all. Evaluation of these strategies in implementation effectiveness trials is warranted.

## Data Availability

Data are available upon reasonable request. The data sets used and/or analysed during the current study are available from the corresponding author on reasonable request.
