# Supplementary material for: Implementation preferences for the management of sexually transmitted infections in the South African health system: a discrete choice experiment
Source: Sex Transm Infect. 2023 Nov 2;100(1):10–6. doi: 10.1136/sextrans-2023-055816 (PMC10850665; doi:10.1136/sextrans-2023-055816)
Supplement: Supplementary data [file sextrans-2023-055816supp001.pdf]

S1 Table. Study questionnaire and DCE tool

Information Session
Before the session starts, we will conduct a brief information session (10mins) explaining what STI's are, types of STI's and current approaches for management.

Socio-Demographics	
1. Date of Birth	Day/Month/Year
2. Gender	1- Male<br>2- Female<br>3- Transgender man<br>4- Transgender woman<br>98- Other, (please specify):<br>___________________________
3. Do you have a mobile phone?	1 – Yes<br>2 – No
4. Do you have access to the internet?	1 – Yes<br>2 – No
5. Employment Status	1 -Employed or self-employed FULL-TIME (at least 40 hours a week)<br>2- Employed or self-employed PART-TIME (less than 40 hours a week)<br>3- Unemployed<br>98- Other (please specify):<br>____________________________
6. Approximately how far away do you live from your preferred clinic?	[Distance in kms]
7. Relationship Status [multiple response options]	1- Married<br>2 - In a stable relationship<br>3 – Casual partner/s<br>4 - Single, no partner/s<br>3- Casual relationships only<br>98- Other (please specify)<br>______________________________
**Sexual & Reproductive Health Behaviour**	
8. Number of Current Sex Partners	1 – One<br>2 – Two to four<br>3 – Five to six<br>4 – Over six
9. Are you having sex with a partner whose HIV status you do not know?	1 – Yes<br>2 - No
10. Have you had unprotected sex in the last year and did not use a condom?	1 – Yes<br>2 – No
11. Are you having sex with partners who are HIV positive?	1 – Yes<br>2 – No<br>3 – Unsure
12. Have you had sex under the influence of alcohol or drugs?	1 – Yes<br>2 – No
13. Have you ever been treated for an STI?	1 – Yes<br>2 – No

Iwuji C, et al. Sex Transm Infect 2024; 100:10–16. doi: 10.1136/sextrans-2023-055816

14. Do you currently have any of these symptoms? [multiple response options]	1- Change in Vaginal Discharge [smell, colour, quantity]<br>2- Lower abdominal pain<br>3- Pain during sex<br>4- Genital sores<br>5- Pain when passing urine<br>6- Urethral discharge [“drops”]
15. Are you on a family planning method?	1 – Yes<br>2 – No
16. If yes to Q15, specific	1 – 3 month injection (Depo-Provera<br>2 – 2 month injection (Nuristerate)<br>3 – Oral Pill<br>4 – Implant<br>5 – IUD<br>6 – Other
17. Are you planning on falling pregnant in the near future?	1 – Yes<br>2 – No<br>3 – Not Applicable
**Service Utilization**	
18. Did you attend a health facility in the past during lockdown periods?	1 – Yes<br>2 – No
19. If no to Q18, why?	1 – Did not require services<br>2 – Concerned about getting infected with Coronavirus<br>3 – In isolation or quarantine due to coronavirus exposure<br>4 – Temporary clinic closure due to coronavirus<br>5 – Difficulties due to movement restrictions<br>6 – Financial Challenges<br>7 – Other
20. If yes, what service did you access?	1 – Services for minor ailments<br>2 – Family Planning<br>3 – PrEP<br>4 – STI diagnosis, care and treatment<br>5 – Care for chronic conditions<br>6 – COVID-19 testing<br>7 – Other
21. Did you receive any service at a community based organization?	1 – Social Grant Collection<br>2 – Feeding Programme<br>3 – Learner Support Programme<br>4 – Gender Based Violence Services<br>5 – Other

Iwuji C, *et al. Sex Transm Infect* 2024; 100:10–16. doi: 10.1136/sextrans-2023-055816

Discrete Choice Experiments

Before the DCE session starts, we will inform the clients about the questions and description of choices as per tool #DCE-002.

Read to client: We will like you to select either option A or B from each of 12 questions below. Select the choice that is most preferred to you. The questionnaire will take you approximately 30-40 minutes to complete.

Question 1

Imagine you were being offered an STI test today, which testing option would you prefer, A or B?

	a	b
**How the STI testing sample is taken**	No sample is taken, care is based only on presenting symptoms	By a health care professional at the clinic
**How and when you are told if you have an STI**	The same day after a 2 hour wait at the clinic by a health care professional	In 1-7 days time via text message (SMS)
**How and when treatment for your STI is given if required**	From a place near you such as a local pharmacy	From a place near you such as a local pharmacy
**How your partner(s) is / are notified if you have an STI**	You notify your partner using a notification slip	Your partner(s) is / are notified directly by a health care professional
**Which option would you prefer, A or B (tick one box)?**		

Question 2

Imagine you were being offered an STI test today, which testing option would you prefer, A or B?

	a	b
**How the STI testing sample is taken**	You self-sample at the clinic	No sample is taken, care is based only on presenting symptoms
**How and when you are told if you have an STI**	The same day after a 2 hour wait at the clinic by a health care professional	The same day after a 4 hour wait at the clinic by a health care professional
**How and when treatment for your STI is given if required**	From a place near you such as a local pharmacy	At the clinic the same day
**How your partner(s) is / are notified if you have an STI**	You notify your partner using a notification slip	Expedited partner treatment
**Which option would you prefer, A or B (tick one box)?**		

3

Question 3

Imagine you were being offered an STI test today, which testing option would you prefer, A or B?

	a	b
**How the STI testing sample is taken**	You self-sample at the clinic	By a health care professional at the clinic
**How and when you are told if you have an STI**	In 1-7 days time via a secure online website	In 1-7 days time via text message (SMS)
**How and when treatment for your STI is given if required**	From a place near you such as a local pharmacy	At the clinic during a follow-up appointment
**How your partner(s) is / are notified if you have an STI**	Your partner(s) is / are notified directly by a health care professional	Expedited partner treatment
**Which option would you prefer, A or B (tick one box)?**		

Question 4

Imagine you were being offered an STI test today, which testing option would you prefer, A or B?

	a	b
**How the STI testing sample is taken**	By a health care professional at the clinic	You self-sample at the clinic
**How and when you are told if you have an STI**	In 1-7 days time via a secure online website	In 1-7 days time via text message (SMS)
**How and when treatment for your STI is given if required**	At the clinic during a follow-up appointment	From a place near you such as a local pharmacy
**How your partner(s) is / are notified if you have an STI**	Expedited partner treatment	You notify your partner using a notification slip
**Which option would you prefer, A or B (tick one box)?**		

Question 5

Imagine you were being offered an STI test today, which testing option would you prefer, A or B?

	a	b
**How the STI testing sample is taken**	By a health care professional at the clinic	You self-sample at the clinic
**How and when you are told if you have an STI**	The same day after a 4 hour wait at the clinic by a health care professional	In 1-7 days time via a secure online website

Iwuji C, *et al. Sex Transm Infect* 2024; 100:10–16. doi: 10.1136/sextrans-2023-055816

**How and when treatment for your STI is given if required**	From a place near you such as a local pharmacy	At the clinic during a follow-up appointment
**How your partner(s) is / are notified if you have an STI**	Your partner(s) is / are notified directly by a health care professional	Expedited partner treatment
**Which option would you prefer, A or B (tick one box)?**		

Question 6

Imagine you were being offered an STI test today, which testing option would you prefer, A or B?

	**a**	**b**
**How the STI testing sample is taken**	You self-sample at the clinic	By a health care professional at the clinic
**How and when you are told if you have an STI**	In 1-7 days time via text message (SMS)	The same day after a 4 hour wait at the clinic by a health care professional
**How and when treatment for your STI is given if required**	At the clinic during a follow-up appointment	At the clinic the same day
**How your partner(s) is / are notified if you have an STI**	Your partner(s) is / are notified directly by a health care professional	You notify your partner using a notification slip
**Which option would you prefer, A or B (tick one box)?**		

Question 7

Imagine you were being offered an STI test today, which testing option would you prefer, A or B?

	**a**	**b**
**How the STI testing sample is taken**	You self-sample at the clinic	By a health care professional at the clinic
**How and when you are told if you have an STI**	In 1-7 days time via text message (SMS)	In 1-7 days time via a secure online website
**How and when treatment for your STI is given if required**	From a place near you such as a local pharmacy	At the clinic during a follow-up appointment
**How your partner(s) is / are notified if you have an STI**	Expedited partner treatment	Your partner(s) is / are notified directly by a health care professional

**Which option would you prefer, A or B (tick one box)?**		

Question 8

Imagine you were being offered an STI test today, which testing option would you prefer, A or B?

	**a**	**b**
**How the STI testing sample is taken**	By a health care professional at the clinic	You self-sample at the clinic
**How and when you are told if you have an STI**	In 1-7 days time via a secure online website	The same day after a 4 hour wait at the clinic by a health care professional
**How and when treatment for your STI is given if required**	From a place near you such as a local pharmacy	At the clinic the same day
**How your partner(s) is / are notified if you have an STI**	Expedited partner treatment	Your partner(s) is / are notified directly by a health care professional
**Which option would you prefer, A or B (tick one box)?**		

Question 9

Imagine you were being offered an STI test today, which testing option would you prefer, A or B?

	**a**	**b**
**How the STI testing sample is taken**	No sample is taken, care is based only on presenting symptoms	By a health care professional at the clinic
**How and when you are told if you have an STI**	The same day after a 2 hour wait at the clinic by a health care professional	In 1-7 days time via a secure online website
**How and when treatment for your STI is given if required**	At the clinic the same day	From a place near you such as a local pharmacy
**How your partner(s) is / are notified if you have an STI**	Your partner(s) is / are notified directly by a health care professional	You notify your partner using a notification slip
**Which option would you prefer, A or B (tick one box)?**		

Question 10

Imagine you were being offered an STI test today, which testing option would you prefer, A or B?

	a	b
**How the STI testing sample is taken**	You self-sample at the clinic	No sample is taken, care is based only on presenting symptoms
**How and when you are told if you have an STI**	The same day after a 4 hour wait at the clinic by a health care professional	The same day after a 2 hour wait at the clinic by a health care professional
**How and when treatment for your STI is given if required**	From a place near you such as a local pharmacy	At the clinic the same day
**How your partner(s) is / are notified if you have an STI**	Expedited partner treatment	You notify your partner using a notification slip
**Which option would you prefer, A or B (tick one box)?**		

Question 11

Imagine you were being offered an STI test today, which testing option would you prefer, A or B?

	a	b
**How the STI testing sample is taken**	You self-sample at the clinic	By a health care professional at the clinic
**How and when you are told if you have an STI**	The same day after a 4 hour wait at the clinic by a health care professional	The same day after a 2 hour wait at the clinic by a health care professional
**How and when treatment for your STI is given if required**	At the clinic the same day	From a place near you such as a local pharmacy
**How your partner(s) is / are notified if you have an STI**	You notify your partner using a notification slip	Your partner(s) is / are notified directly by a health care professional
**Which option would you prefer, A or B (tick one box)?**		

Question 12

Imagine you were being offered an STI test today, which testing option would you prefer, A or B?

	a	b

Iwuji C, *et al. Sex Transm Infect* 2024; 100:10–16. doi: 10.1136/sextrans-2023-055816

**How the STI testing sample is taken**	By a health care professional at the clinic	You self-sample at the clinic
**How and when you are told if you have an STI**	In 1-7 days time via text message (SMS)	The same day after a 2 hour wait at the clinic by a health care professional
**How and when treatment for your STI is given if required**	At the clinic during a follow-up appointment	At the clinic the same day
**How your partner(s) is / are notified if you have an STI**	You notify your partner using a notification slip	Expedited partner treatment
**Which option would you prefer, A or B (tick one box)?**		

Iwuji C, *et al. Sex Transm Infect* 2024; 100:10–16. doi: 10.1136/sextrans-2023-055816

S1_Figure - Pictorial Illustrations

AAA

brighton and sussex medical school

University of the Witwatersrand WITS RHI

Information before completing the next survey

The next part of the survey involves you selecting from a list of options. Before you move to the next part of the survey, we would like to run you through the response options. You can either read the table below or listen to the audio recording provided. Once you have read or listened to the response options, press the 'Go to survey' button below to access the survey.

To listen to the audio recording, please press the play button below.

0:00 / 2:48

Information on Response Options

IMPORTANT: PLEASE TAKE TIME TO UNDERSTAND THIS INFORMATION BEFORE PROCEEDING TO THE SURVEY

		OPTIONS	DESCRIPTION
**How the STI testing sample is taken**	A.	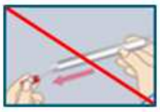 No sample is taken, care is based only on presenting symptoms	No samples are taken – you are given treatment just based on whether you have symptoms or not
	B.	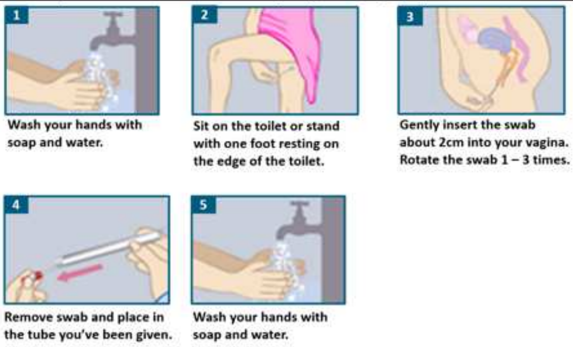 You self-sample at the clinic	There are different types of samples – urine for men and vaginal swab for women [Display swab]. After being provided with instructions, you could take the sample yourself at a private place at the clinic
	C.	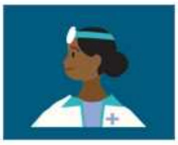 By a health care professional at the clinic	The provider takes a sample when he/she is examining you

		OPTIONS	DESCRIPTION
**How and when you are told if you have an STI**	A.	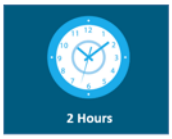 The same day after a 2 hour wait at the clinic by a health care professional	This may be the shortest time you will spend in the clinic in total because diagnosis is based on symptoms only, no samples are taken so the provider will not know whether you really have an infection or not and will also not know which infection/s you have
	B.	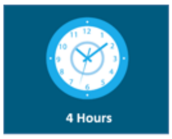 The same day after a 4 hour wait at the clinic by a health care professional	You will spend a longer time in the clinic because samples will be taken and tested at clinic so you will definitely know if you have an infection and what it is
	C.	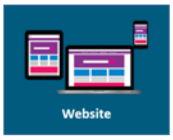 In 1-7 days time via a secure online website	You may spend about 2 hours in the clinic because samples will be taken but it will not be tested at the clinic but rather they will be sent away for testing so your results will not be available on the same day. You will be given a secure code at the clinic and a website address to check your results privately. If you have an infection you may still have to go back to the clinic for treatment.
	D.	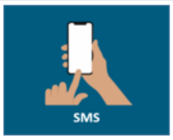 In 1-7 days time via text message (SMS)	You may spend about 2 hours in the clinic because samples will be taken but it will not be tested at the clinic but rather they will be sent away for testing so your results will not be available on the same day. You will receive a text message when your results are ready which will either say [Your Results Are Ready – No Follow-up Visit Required] or [Your Results Are Ready – Please present at your preferred clinic as soon as possible]. If you have an infection you will have to go back to the clinic for treatment.

		OPTIONS	DESCRIPTION
**How and when treatment for your STI is given if required**	A.	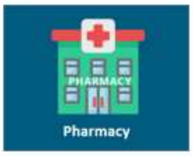<br>From a place near you such as a local pharmacy	Medication can be picked up from a local pharmacy
	B.	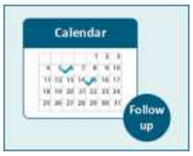<br>At the clinic during a follow-up appointment	If you receive a SMS requiring you to revisit the clinic or if the online system indicates you have an infection, you will be required to come back to the clinic to collect your treatment
	C.	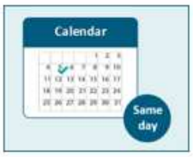<br>At the clinic the same day	If you are being treated according to your symptoms or not or if are being tested and the test in being done at the clinic on the same day

		OPTIONS	DESCRIPTION
**How your partner(s) is / are notified if you have an STI**	A.	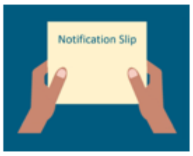<br>You notify your partner using a notification slip	We will give you a slip to take to your partner/s. The slip will inform your partner to attend the clinic for diagnosis and/or treatment.
	B.	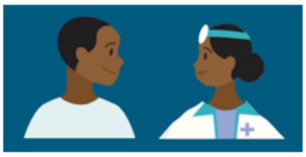<br>Your partner(s) is / are notified directly by a health care professional	If you prefer, the provider can contact your partner and invite them to the clinic for testing and treatment, without mentioning you
	C.	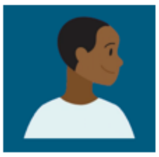<br>Expedited partner treatment	If you are being treated for an infection you will be given treatment to give to your partner/s for the same infection.

Submit

S2 Table: Predictors of class membership

**Predictors of Group 1 (self-led) membership; Group 2 reference**	**Odds ratio (95% CI); p value**
Gender (F vs M)	0.69 (0.29-1.66); p=0.405
Age (>25years vs <25 years)	4.20 (1.77 -9.93); p=0.001
STI symptoms (N vs Y)	0.93 (0.80 – 1.09); p=0.390
Previous STI treatment (N vs Y)	1.69 (0.74 – 3.85); p=0.206
Clinic used for care (Peri-urban vs Urban)	5.17 (1.46 – 18.24); p=0.011
Clinic used for care (Other vs. Urban)	2.62 (0.90 -7.60); p=0.076
Data collection method (completion with support vs self-completion)	0.54 (021-1.39); p=0.200
Employment (Employed vs not employed)	0.35 (0.13 – 0.94); p=0.038
Employment (student vs not employed)	0.57 (0.18-1.76); p=0.327
